# Beyond host defense and tissue injury: the emerging role of neutrophils in tissue repair

**DOI:** 10.1152/ajpcell.00652.2023

**Published:** 2024-01-08

**Authors:** Salma A. Rizo-Téllez, János G. Filep

**Affiliations:** Department of Pathology and Cell Biology, University of Montreal and Research Center, Maisonneuve-Rosemont Hospital, Montreal, Quebec, Canada

**Keywords:** inflammation, neutrophil heterogeneity, resolution of inflammation, tissue injury, tissue repair

## Abstract

Neutrophils, the most abundant immune cells in human blood, play a fundamental role in host defense against invading pathogens and tissue injury. Neutrophils carry potentially lethal weaponry to the affected site. Inadvertent and perpetual neutrophil activation could lead to nonresolving inflammation and tissue damage, a unifying mechanism of many common diseases. The prevailing view emphasizes the dichotomy of their function, host defense versus tissue damage. However, tissue injury may also persist during neutropenia, which is associated with disease severity and poor outcome. Numerous studies highlight neutrophil phenotypic heterogeneity and functional versatility, indicating that neutrophils play more complex roles than previously thought. Emerging evidence indicates that neutrophils actively orchestrate resolution of inflammation and tissue repair and facilitate return to homeostasis. Thus, neutrophils mobilize multiple mechanisms to limit the inflammatory reaction, assure debris removal, matrix remodeling, cytokine scavenging, macrophage reprogramming, and angiogenesis. In this review, we will summarize the homeostatic and tissue-reparative functions and mechanisms of neutrophils across organs. We will also discuss how the healing power of neutrophils might be harnessed to develop novel resolution and repair-promoting therapies while maintaining their defense functions.

## Graphical abstract

**Figure:**
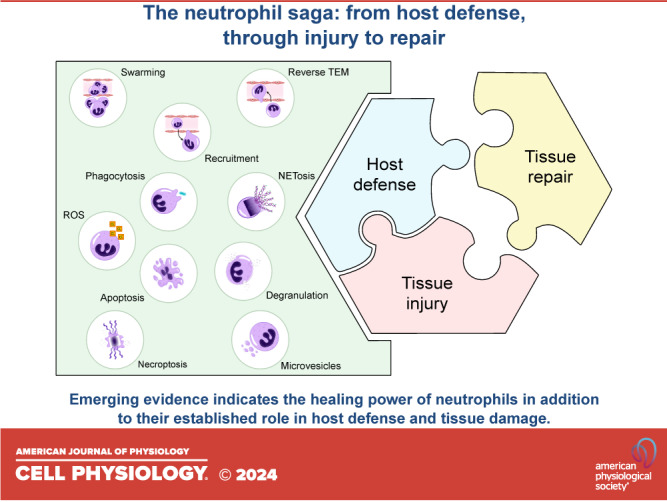

## INTRODUCTION

Neutrophils are the most abundant immune cells in human blood and play a pivotal role in defense against invading pathogens and tissue injury. Neutrophils are rapidly recruited to the affected site where they deploy an impressive array of mechanisms that contribute to the elimination of the invading pathogens and necrotic tissue (1–3). This ideally prompts repair of tissue damage and return to homeostasis (4). However, the neutrophils’ powerful weaponry to combat pathogens is capable of inflicting damage to the host and prolongs the initial response through feed-forward inflammatory mechanisms (5, 6). Indeed, neutrophilia, dysregulated neutrophil trafficking, and activation are common features of diverse pathological conditions (1, 7). Consistently, numerous studies have documented the beneficial effects of neutrophil depletion or blockade of neutrophil function in preclinical models (8). The prevailing and rather simplistic view of the neutrophils emphasizes the dichotomy, antibacterial defense versus tissue damage, of their function. It is well known that neutropenic patients are susceptible to infections (9), which are associated with tissue damage and poor outcomes (10), implying contribution of neutrophils to tissue repair. Accumulating evidence indicates a complex, likely organ-, and context-dependent homeostatic and repair functions for neutrophils (11, 12). In this review, we focus on the emerging role of neutrophils in contributing to inflammation resolution and tissue repair. We also discuss organ-specific mechanisms and how these might be harnessed to develop novel resolution-promoting therapies.

## NEUTROPHILS IN HOMEOSTASIS

The abundance and short half-life of blood neutrophils indicate a massive cellular turnover (2). The bone marrow neutrophil lineage is composed of the hematopoietic stem cell pool, the mitotic pool, and the postmitotic pool, which comprises metamyelocytes, band cells, and mature neutrophils (13). Egress of mature neutrophils from the bone marrow to the blood is regulated by the cytokine granulocyte colony-stimulating factor (G-CSF) (14) and the chemokine CXC-motif ligand 1 (CXCL1) and CXCL2 (15). As part of the neutrophil feedback loop, “aged” neutrophils (CD62L^low^, CXCR4^high^) regress from the blood into the bone marrow, where they are phagocytosed by resident macrophages via the cholesterol-sensing nuclear liver receptor X pathway (16, 17). Neutrophil clearance in the bone marrow facilitates egress of hematopoietic progenitor cells into the circulation through downregulation of CXCL12 in the stroma, thereby reducing the bone marrow hematopoietic niche (13, 16). In mice, circadian oscillations in the number of hematopoietic stem progenitor cells (16), a neutrophil-autologous timer, consisting of clock-related genes (such as brain and muscle Arnt-like 1, *Bmal1,* and *Clock*), and the C-X-C chemokine receptor type 2 (CXCR2) signaling pathway (18), and the microbiome (19) control the proportion of “aged” neutrophils. Neutrophil clearance may also occur in peripheral tissues, leading to reduction of circulating levels of G-CSF and interleukin 17 A (IL-17A), which in turn, promote retention of hematopoietic progenitor cells within the bone marrow, thereby maintaining hematopoietic activity (11, 20). While “aging” is thought to favor neutrophil clearance (18), other studies reported a role for “aged” neutrophils in the first line of defense in acute inflammation (21). Neutrophils returning into the bone marrow can temporarily be engulfed by megakaryocytes without phagocytosis (termed emperipolesis), transfer membrane fragments to newly produced platelets, and thus modulate thrombopoiesis (22). The fate of neutrophils that underwent emperipolesis remains to be investigated.

The hematopoietic system rapidly adapts to a higher demand for neutrophils during severe acute infections by switching from steady state to emergency granulopoiesis, critical for host survival (23, 24). Pathogen sensing by nonhematopoietic and hematopoietic progenitor cells initiates de novo production of neutrophils through the release of G-CSF, chemokine C-X-C motif ligand 12 (CXCL12), and chemokine CC motif ligand 3 (CCL3) in the bone marrow (and extramedullary sites) and mobilization of mature neutrophils from the intravascular (marginated) pools (23, 24). Pathogen sensing also leads to a switch from the transcription factor CCAAT-enhancer-binding protein-α (C/EBPα), the master regulator of steady-state granulopoiesis, to C/EBPβ to drive emergency granulopoiesis (25, 26), characterized by an increased egress of immature neutrophils, myelocytes, metamyelocytes, and band cells into the circulation. Whole blood single-cell multiomic mapping indicated altered granulopoiesis and identified CD66b^+^ immunosuppressive mature and immature neutrophils in patients with sepsis, which were associated with poor outcomes (27). The extreme sepsis response endotype is characterized by higher frequencies of interleukin-1 receptor type 2-expressing (IL1R2^+^) immature neutrophils, epigenetic and transcriptomic signatures of emergency granulopoiesis in hematopoietic stem and progenitor cells, and signal transducer and activatior of transcription 3 (STAT3)-mediated gene expression signature (27). In systemic juvenile idiopathic arthritis, the percentage of immature neutrophils (banded neutrophils and granulocyte precursors) is increased and reflected by the higher proportion of CD16^dim^ and CD62L^low^neutrophils (28, 29). Higher numbers of hypersegmented neutrophils in the blood and appearance of a unique neutrophil population that can present antigens identify the active phase and progression of the disease (28, 30, 31). Severe acute respiratory syndrome coronavirus 2 (SARS-CoV-2) infection resulted in dramatic increases in the number of immature neutrophils, which strongly correlated with disease severity (32).

The mechanisms that orchestrate the return from emergency to steady-state conditions are incompletely understood but are known to involve suppressor of cytokine signaling proteins (23). Recent results indicate that resolvin D4 (RvD4), generated from n-3 docosahexaenoic acid (33) at distant sites of bacterial infection, exerts multipronged actions to promote the return to steady-state in mice (34). RvD4 prevents leukotriene B_4_ (LTB_4_)-stimulated neutrophil deployment and facilitates the removal of aged neutrophils by the bone marrow (34). Furthermore, RvD4 disengages emergency granulopoiesis, as evidenced by downregulation in granulocyte lineage trajectory in LSKs (Lineage^-^Sca^ + ^cKit^+^), granulocyte monocyte progenitors, preneutrophil and immature neutrophils without affecting the trajectory of terminally differentiated cells, mature neutrophils, and circulating neutrophils (34).

Studies using neutrophil-specific reporter mice revealed that in steady state, neutrophils infiltrate most tissues and may influence homeostatic functions (11). For example, neutrophils are bone fide resident cells in the lymph node in humans and mice (35). Live imaging identified two populations of resident neutrophils: Ly6G^high^ mature neutrophils, which continuously patrol uninfected human and mouse lymph node (36), and stationary Ly6G^low^ immature neutrophils, which rapidly mature and mobilize to boost tissue defense during bacterial infections (37, 38). Another subset, B-cell helper neutrophils have been found in the perifollicular zones of the human spleen (39), though this has been disputed (40). Spleen neutrophils stimulated IgM secretion in vitro by inducing the secretion of B-cell activating factor (BAFF), IL-21, and pentraxin 3 (41). Pentraxin 3 also increased IgG production after infection with blood-borne encapsulated bacteria (42).

Under steady state, intrahepatic neutrophil counts oscillate throughout the day, being the highest during the resting phase and the lowest during the active phase in mice (43), coinciding with lipogenesis in hepatocytes (44). Infiltrating neutrophils release neutrophil elastase that through facilitating expression of the clock genes *Bmal1* and *Clock* promotes mouse hepatocytes to become lipogenic (43). Similar changes were detected in humans, raising the possibility of neutrophil regulation of daily hepatic metabolism (43). Clock dysfunction accelerates the development of various liver diseases, which, in turn, disrupt clock function, forming a deleterious feed-forward amplification loop (44).

Recent studies indicate that during prenatal development, neutrophil-produced 12-hydroxyeicosatetraenoic acid (12-HETE) imprints the long-term self-renewing program of alveolar macrophages (45). The origin and phenotype of neutrophils in the prenatal lung are not known. As neutrophil function declines with age (19), it is tempting to speculate that their reduced capacity to produce 12-HETE might contribute to aging-related decrease in the number of alveolar macrophages (46), thereby increasing susceptibility to pulmonary infection in the elderly.

## THE NEUTROPHIL PARADOX: HOST DEFENSE VERSUS TISSUE DAMAGE

**Figure 1.**
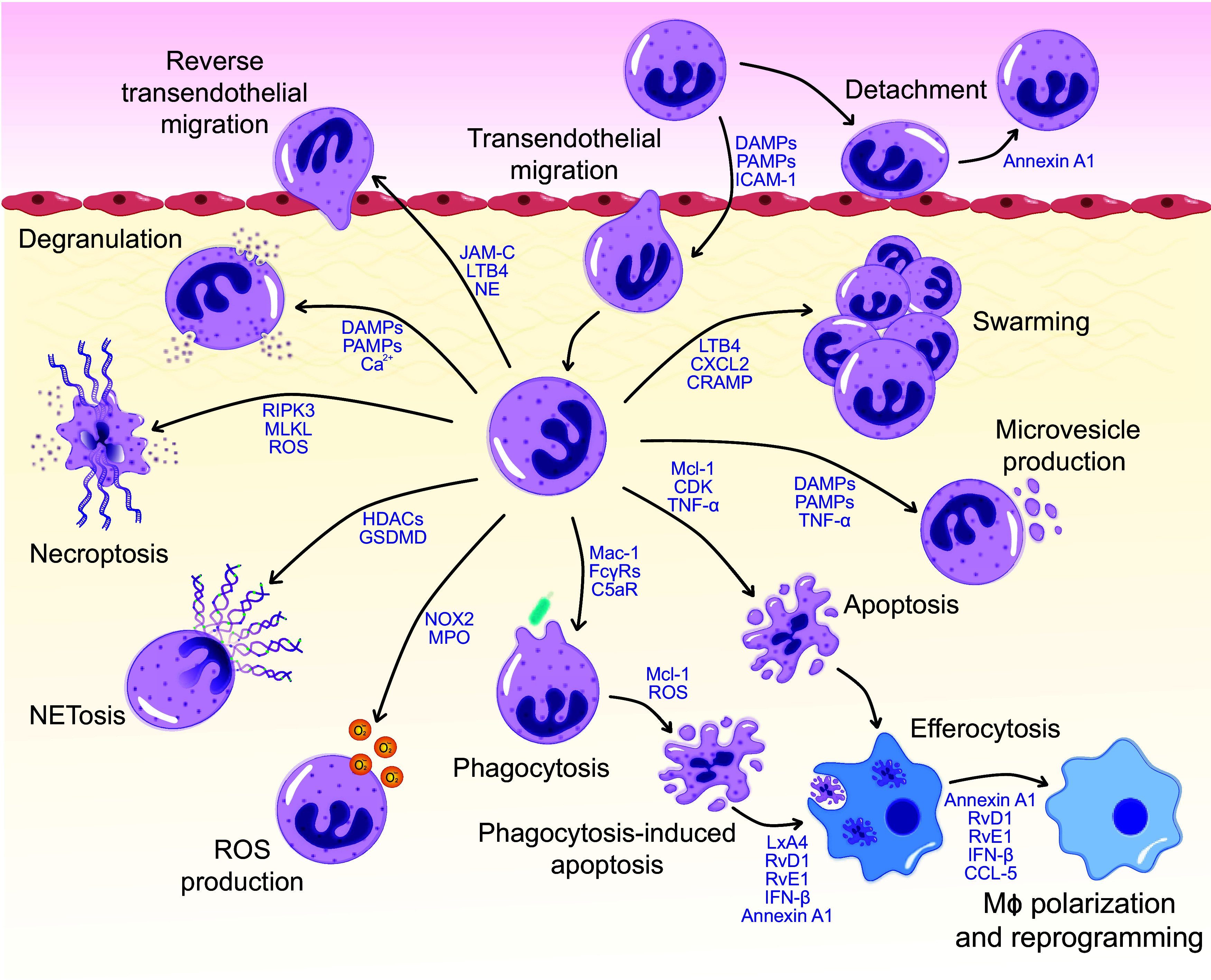
Neutrophil kinetics and functions in inflamed tissues. Neutrophils are rapidly recruited from the blood to the sites of infection or injury through a multistep process, involving rolling along and adhesion to the endothelium followed by transendothelial migration. Mobilization of annexin A1 to the neutrophil surface functions as a repellent signal, resulting in detachment of adherent neutrophils. Neutrophils swarm toward and form clusters around pathogens. Emigrated neutrophils may egress from the tissue through reverse transendothelial migration. Reversed transmigrated neutrophils may return to the bone marrow for destruction or may disseminate the infection and cause distant organ damage. Neutrophil may trap and destroy bacteria through phagocytosis, degranulation, or release of extracellular traps (NETs). Phagocytosis induces neutrophil apoptosis. Neutrophil lifespan is also affected by DAMPs, PAMPs, and other mediators present in the inflammatory microenvironment. Apoptotic neutrophils express “find-me” and “eat-me” signals and are removed by macrophages via efferocytosis. This would lead to polarization and reprogramming of macrophages toward a proresolution phenotype. Neutrophils could also undergo programmed necrosis (necroptosis) concomitant with or independent from NET extrusion. The molecular switches that govern execution of intracellular or extracellular bacterial killing mechanisms are still poorly characterized. Neutrophils may communicate with neighboring cells and remodel tissue matrix through ROS production and release of granule constituents or microvesicles. Selected effector molecules are shown for each process. C5aR, complement C5a receptor; CCL5, chemokine (C-C motif) ligand 5; CDKs, cyclin-dependent kinases; CRAMP, cathelin-related antimicrobial peptide; CXCL2, chemokine (C-X-C motif) ligand 2; DAMPs, damage-associated molecular patterns; FcγRs, IgG receptors; GSDM D, gasdermin D; HDACs, histone deacetylases; ICAM-1, intercellular adhesion molecule 1; IFN-β, interferon-β; JAM-C, junctional adhesion molecule; LTB4, leukotriene B4; MLKL, mixed lineage kinase domain-like protein; NE, neutrophil elastase; NOX2, nitric oxide synthase 2; Mcl-1, myeloid leukemia 1; MPO, myeloperoxidase; PAMPs, pathogen-associated molecular patterns; RIPK3, receptor-interacting protein kinase 3; ROS, reactive oxygen species; RvD1, resolvin D1; RvE1, resolvin E1; TNF-α, tumor-necrosis factor-α. Modified from Filep and Ariel (49).

Infection or damage triggers a rapid response mediated by the tissue itself and elements of the innate immune system, including neutrophils, which lead to acute inflammation. Neutrophils carry a lethal arsenal of weaponry to the perturbed site to eliminate invading pathogens. Undoubtedly, this reaction may inflict collateral tissue injury during the initial phase of the inflammatory reaction (47), but it eventually leads to complete repair (4). Once neutrophils have fulfilled their physiological defense function, they must be “switched off” and removed from the affected site during the resolution phase. Considering their lethal armaments, excessive recruitment or failure of their timely removal, neutrophils have considerable potential to perpetuate tissue damage. Indeed, preclinical and clinical data support a pivotal role for neutrophils in the pathogenesis of various chronic inflammatory conditions, including atherosclerosis, metabolic syndrome, inflammatory bowel disease, autoimmune disease, severe asthma, and cancer (5, 48). There is a growing recognition of the complexity and the dual role of neutrophils. These cells exhibit phenotypic heterogeneity and functional versatility, which shape the outcome of the inflammatory response and ultimately determine whether neutrophils will act as a friend or foe (Fig. 1).

### Multidirectional Migration

Neutrophil trafficking into damaged tissues is not only vital for host defense but also relevant in pathological conditions. Neutrophils express a multitude of receptors that allow detecting pathogen-associated molecular patterns (PAMPs) and damage-associated molecular patterns (DAMPs), which initiate their recruitment into the inflammatory locus. The neutrophil recruitment cascade is modeled as a tightly orchestrated multistep process and has been detailed in excellent reviews (50–53). Recent work showed that in addition to certain common selectin and β_2_ integrin-dependent steps, neutrophil recruitment also relies on tissue-specific anatomic properties and molecular patterns, shaping a controlled inflammatory response (53). Neutrophil recruitment not only appears mainly in postcapillary venules within the microcirculation but also occurs within small capillaries in the lung (54) and arteries as exemplified in the case of atherosclerosis (48). Neutrophil trafficking into the lung and microbiome-mediated neutrophil recruitment into the intestine rely on CXC-motif chemokine receptor 2 (CXCR2) (53, 55). In the liver, recruitment appears mostly selectin-independent through CD44/hyaluronan-dependent interactions (56), whereas caspase recruitment domain-containing protein 9 (CARD9) has been implicated in neutrophil trafficking into the joints (53). In experimental stroke, neutrophil invasion of the brain depends on very late antigen 4 (VLA-4) (57). These findings offer potential therapeutic targets for tissue-specific modulation of neutrophil recruitment, though development of such approaches is rather challenging.

Relocation of annexin A1 (AnxA1) from the cytoplasm pool to the cell surface results in detachment of already adhered neutrophils (58, 59). Lipoxin A_4_ (LXA_4_) evokes mobilization of AnxA1 and acts in concert with AnxA1 through formyl-peptide receptor 2 (FPR2, which also binds LXA_4_, hence termed as ALX/FPR2) (60) to dampen neutrophil recruitment into inflamed tissues as shown in ischemic mesenteric postcapillary venules (59) and atherosclerotic lesions (61, 62). AnxA1-deficient mice exhibit enhanced neutrophil transmigration (63), underscoring the importance of AnxA1 controlling of neutrophil detachment. The function and fate of detached neutrophils remain to be studied.

Two-photon intravital microscopy studies have revealed a coordinated simultaneous migration of large numbers of neutrophils, referred to as “neutrophil swarming,” in diverse tissues under infectious and sterile conditions (64). Neutrophil swarming occurs following transmigration from capillary vessels, where they form small transient clusters and subsequently larger clusters, which can insulate the affected site from the surrounding healthy tissue (65–67). G-CSF enhances swarming and neutrophil ability to restrict growth of the fungal pathogen *Candida albicans* (68), however, formation of larger clusters around *Candida* can lead to blockage of blood flow in the pulmonary vasculature of infected mice (69). Swarming requires LTB_4_ produced by neutrophils, which signals through the LTB_4_ receptor 1 (BLT1) and the β_2_ integrins Mac-1 and LFA-1 (67). Microscale arrays identified numerous protein mediators, including CXCL8, galectin-3, and pentraxin-3, which modulate LTB_4_-driven swarming (70). Pharmacological blockade of BLT1 prevented neutrophil swarming and vascular occlusion without affecting neutrophil microbial killing (69).

There is evidence that neutrophils can move away from the inflamed area back to the vascular lumen, known as reverse transendothelial migration (TEM) (71–73). This phenomenon was demonstrated in in vitro models (72), zebrafish (71), mouse cremaster venules (74), and liver (75), and appears to be most prevalent under ischemia-reperfusion injury. Reverse TEM depends on the enzymatic degradation of the junctional adhesion molecule C (JAM-C) by Mac-1-bound neutrophil elastase (74, 76). Ischemia-reperfusion triggers formation of LTB_4_, which through BLT1 evokes release of neutrophil elastase (76). Although the chemotactic cues are largely unknown, CXCL8a/CXCR2 signaling was found to promote reverse TEM (77), whereas CXCL8 (IL-8) functions as a chemorepellent (78). Reversely transmigrated neutrophils display a distinct phenotype (ICAM1^high^/CXCR1^low^) and enhanced oxidative capacity (72, 74), upregulate CXCR4 during their passage through lung capillaries, facilitating their homing back to the bone marrow (75). Thus, reverse TEM may either contribute to removal of neutrophils from the injured tissue, thereby limiting the inflammatory reaction (52) or disseminate inflammation and contribute to distant organ injury. Indeed, ICAM1^high^/CXCR1^low^ neutrophils were detected in the blood and the lung vasculature and correlated with the severity of lung injury in patients and mice with acute pancreatitis (79).

During pathogen-driven inflammation, neutrophils were found to emigrate from the tissue through the lymphatic vessels and shuttle live bacteria to the draining lymph node (80, 81). Migrating neutrophils exhibit a distinct phenotype (CD11b^high^, CD62L^low^, CXCR2^low^), major histocompatibility complex class II, and the costimulatory molecules CD80 and CD86, enabling them to present antigens to the adaptive immune system (81, 82). Neutrophils may enter the lymph nodes through high endothelial venules, involving L-selectin interaction with peripheral node addressin (PNAd) (35, 83) and/or afferent lymphatic vessels (36). A portion of neutrophils was found to recirculate within 24 h through the efferent lymphatic vessels along a sphingosine-1-phosphate gradient (83).

### Functional Versatility

Neutrophils patrol and protect the host from invading pathogens while preserving tissue integrity, however, they are capable of inflicting damage to the host. The molecules and effector mechanisms underlying the dual function of neutrophils have received considerable attention and have been reviewed in detail (2, 47, 49, 84–86).

Release of toxic effectors, such as reactive oxygen species (ROS), myeloperoxidase (MPO), proteolytic and antimicrobial proteins can directly damage the endothelium (87) and destroy existing tissue architecture (84, 88). In addition, MPO or MPO-derived oxidants may function as signaling molecules to modulate neutrophil trafficking (89), activation (90, 91) and lifespan (91), and may trigger autoimmunity (92). For example, NADPH oxidase controls neutrophil LTB_4_ generation that drives excessive neutrophilic inflammation in chronic granulomatous disease (93). MPO binds to Mac-1 and evokes MPO release, thereby triggering a feed-forward loop to regulate neutrophil activation and phagocytosis, delay constitutive apoptosis, and perpetuate ongoing tissue destruction (91, 94). Other studies reported that MPO protected mice from lethal endotoxemia (95), possibly through reducing plasma levels of cysteinyl leukotrienes (96).

#### Phagocytosis.

Mature neutrophils are short-lived immune cells in circulation (97, 98), albeit this has been debated (99), and die via apoptosis (100). In the inflamed tissues, their lifespan is increased through delaying constitutive apoptosis in response to PAMPs, DAMPs, and environmental cues, such as hypoxia and extracellular acidosis, though there is no consensus yet regarding their longevity in tissues (49, 98). Increased lifespan would allow neutrophils to undergo phenotypic and functional changes, contributing to their functional versatility (101). Delayed neutrophil apoptosis is a common feature of inflammatory pathologies, including sepsis (102, 103), acute respiratory distress syndrome (104), severe asthma (105), and acute coronary syndrome (106). In experimental models, suppressing neutrophil apoptosis perpetuates tissue damage (91, 107, 108), whereas genetic deletion or pharmacological blockade of cyclin-dependent kinases 5 and 9 (109, 110), 15-epi-LXA_4_ (111), or interferon-β (IFN-β) (112) counter survival signals and accelerate neutrophil apoptosis and the resolution of inflammation.

Neutrophils are potent phagocytes, though neutrophil subsets differ in their phagocytic capacity (3, 113). Phagocytosis of complement or IgG-opsonized targets occurs within minutes, followed by the fusion of specific granules with the phagosome, leading to the destruction of the ingested cargo (3). Complement-mediated phagocytosis is governed by a delicate balance between Mac-1 and the complement C5a receptor (C5aR or CD88) (114, 115) and generates signals to accelerate neutrophil apoptosis (termed as phagocytosis-induced cell death or PICD) (116). Conversely, reduced Mac-1 expression, genetic deletion of C5aR or Toll-like receptor 9 (TLR9) activation-induced neutrophil elastase, and proteinase 3-mediated shedding of C5aR were shown to impair phagocytosis (114, 117), resulting in inefficient bacterial clearance and prolonged lung injury in mice (117). The proresolving lipid mediators produced during the resolution phase of inflammation, resolvin E1 and resolvin D5, which signal through the LTB_4_ receptor BLT1 (118) and G protein-coupled receptor 32 (GPR32) (119), respectively, can enhance phagocytosis of bacteria by naïve mature neutrophils. Furthermore, by preventing TLR9 activation-evoked C5aR shedding, aspirin-triggered 15-epi-LXA_4_ and 17-epi-RvD1, which signal through ALX/FPR2, restored defective phagocytosis, bacterial killing, phagocytosis-induced cell death and consequently accelerated the resolution of *E. coli*-evoked lung injury (117). The fungal pathogen *Candida albicans* can synthesize RvE1 to modulate host immune functions (120). However, treatment with exogenous RvE1 enhanced phagocytosis of Candida by human neutrophils and stimulated clearance of the fungus from blood in a mouse model of systemic candidiasis (120).

Neutrophil phagocytosis is one of the mechanisms pathogens have evolved to escape host defense. Thus, neutrophils may serve as “Trojan horses” that permit dissemination of microorganisms, such as *Toxoplasma gondii* or *Leishmania donovani*, which they cannot destroy, on macrophage engulfment (66, 121). The coccobacillus *Francisella tularensis,* which can evade phagosomal destruction, replicates in the cytosol, sustains mitochondrial integrity, and delays neutrophil apoptosis (122, 123). Continued accumulation of dysfunctional neutrophils at the infection site is thought to contribute to disease exacerbation.

#### NETosis.

Beyond phagocytosis, neutrophils employ neutrophil extracellular traps to capture and destroy microorganisms extracellularly (124). Extensive literature exists on the formation and role of NETs in host defense and inflicting tissue damage (124–126). NETs, released in NADPH oxidase-dependent fashion (known as suicidal NETosis) (127), or in the absence of cellular suicide (named as vital NETosis) (128, 129), effectively capture and destroy a large range of microbes and demarcate the infected area (130, 131). Excessive or uncontrolled NETosis maintains a proinflammatory and prothrombotic environment that underlies numerous pathologies, such as acute respiratory distress syndrome associated with sepsis (132) or SARS-CoV-2 (133–135). Release of NETs, ROS, and proteases inactivate plasma antiproteases that protect against the effects of neutrophil proteases (136), creating a vicious cycle to propagate tissue destruction. Likewise, tumor cells priming neutrophils to release NETs (137) may form an amplifying loop, favoring tumor growth, invasion, and metastases (138, 139).

The molecular switches that trigger phagocytosis, degranulation, or NETosis are still incompletely understood, though it is conceivable that selective activation of these mechanisms assures the most effective neutrophil response to an insult. ALX/FPR2 has been suggested as one of the possible checkpoints for genetic deletion of Fpr2 (the equivalent of human ALX/FPR2) as found to result in excess NET production and more severe lung injury following bacterial infection in mice (132). NETs are degraded by macrophages and dendritic cells, involving both intra- and extracellular processes (140). The antibacterial proteins LL-37 (140) and T-series resolvins, present in resolution exudates (141), were found to facilitate NET uptake by macrophages. Furthermore, LL-37 protected NETs against degradation by bacterial nucleases (140).

#### Efferocytosis.

Removal of apoptotic neutrophils (and other cell types) by macrophages via efferocytosis is essential for terminating the inflammatory reaction. Apoptotic cells express “find-me” signals, such as ATP and UTP, to attract monocytes/macrophages to their vicinity (142) and “eat-me” signals, such as phosphatidylserine, that allow recognition and engulfment (143, 144). Apoptotic cells release lactoferrin, which functions as a “keep-out” signal, i.e., it inhibits granulocyte trafficking without hindering monocyte recruitment, thereby limiting inflammation (145). Efferocytosis reprograms macrophages from the M1 inflammatory phenotype to the M2 anti-inflammatory phenotype (143, 144) and subsequently to a CD11b^low^ proresolution subset (146). CD11b^low^ macrophages exhibit minimal phagocytic activity and produce IFN-β (112, 146), which orchestrates bidirectional cross talk between neutrophils and macrophages to facilitate neutrophil apoptosis and efferocytosis and to accelerate resolution (112).

#### Necroptosis.

Human neutrophils primed with granulocyte-monocyte colony-stimulating factor (GM-CSF) were found to undergo necroptosis (programmed necrosis) following ligation of adhesion receptors through activation of the receptor-interacting protein kinase 3 (RIPK3)-mixed lineage kinase domain-like protein (MLKL) and the mitogen-activated protein kinase (MAPK)-phosphoinositide 3-kinase (PI3K) signaling pathways and subsequent generation of ROS (147, 148). Exposure of neutrophils to monosodium urate crystals (149) or phagocytosis of methicillin-resistant *Staphylococcus aureus* (150) can induce necroptosis. Necroptosis may allow release of ingested bacteria that have survived within the phagosome, leading to persistent infection (151). Necrotic cells release a myriad of DAMPs that function as “find-me” signals and express “eat-me” ligands (partially overlapping with equivalent apoptotic signals) that facilitate their clearance (152, 153). Indirect evidence suggests that neutrophil necroptosis may occur in patients with cutaneous vasculitis, psoriasis, or ulcerative colitis (147), though the relevance of this process to disease progression and to the fate of necroptotic neutrophils remain elusive.

#### Microvesicles.

In addition to surface molecules and humoral mediators, neutrophils actively communicate with surrounding cells through the release of medium-sized extracellular vesicles, called microvesicles or ectosomes (154–156). Neutrophils can release microvesicles in response to a variety of stimuli, including PAMPs or proinflammatory mediators. Neutrophil microvesicles typically express CD66b, CD11b, CD18, MPO and to varying extent phosphatidylserine on their surface (157), but are heterogeneous in composition (158, 159), likely reflecting the activation state of the parent cell. For example, microvesicles generated from adherent neutrophils express the anti-inflammatory proresolution protein AnxA1 on their surface (160), whereas Mac-1 ligation and clustering evoke the release of proinflammatory, antibacterial microvesicles (161). Consistent with the heterogeneity in composition, neutrophil microvesicles likely exert divergent and cell type-selective actions. For example, microvesicles were shown to regulate coagulation (159), induce activation of endothelial cells (162), exert antibacterial actions (154), and anti-inflammatory effects on neutrophils and monocytes/macrophages (156, 163). Another study reported resolution of gout by neutrophil microvesicles through inhibiting complement C5a (C5a)-mediated priming of the inflammasome in mice (155).

### Phenotypic Heterogeneity

Accumulating evidence indicates that neutrophil subpopulations or polarization states exist under steady state and in inflammation, challenging the classical view of neutrophils as a homogeneous population with well-defined and conserved functions (164). Several approaches have been used to define phenotypic and functional heterogeneity in vivo, including nuclear appearance (band cells, mature, and hypersegmented neutrophils), density, single-cell sequencing, and multi-omics analysis of neutrophils from different tissues under steady state, infection, or inflammation (101, 165–167). Cues from distant sites or the inflammatory microenvironment shape different stages of neutrophil activation, which can partly explain their heterogeneity (101, 167–169). However, the mechanisms underlying diversification of mature neutrophils are still incompletely understood (166, 167, 169), and correlating phenotypes with defense, injury, or repair functions is rather challenging.

Circulating human neutrophils to varying percentages express various surface and granule markers under steady state (Table 1). For instance, in steady state, about half of circulating human neutrophils express the glycoprotein NBI (CD177), coexpressing membrane-bound proteinase-3, which facilitates transendothelial migration (174), but may trigger an autoimmune disease, antineutrophil cytoplasmic antibody (ANCA)-dependent vasculitis (176). Smaller portions of neutrophils express olfactomedin-4 (OLFM-4), which may regulate the inflammatory reaction to bacterial infections (206) or evoke ANCA vasculitis (180). Increases in the percentage of OLFM-4^+^ neutrophils were associated with increased mortality in patients with sepsis and acute respiratory distress syndrome (179, 207). Few percentages of blood neutrophils express T-cell receptor-like immunoreceptors with declining repertoire diversity in old age (208), though the function of these receptors is still unknown. Another neutrophil subset is commonly referred to as “angiogenic neutrophils” (CD49d^+^VEGFR1^high^CXCR4^high^), comprising ∼3% of circulating neutrophils in steady state, but accumulate in hypoxic tissues (182, 183).

**Table 1.** Selected neutrophil subsets and their functions in homeostasis and pathological conditions

Phenotype Markers	Species	Model	Function	Reference
CXCR4^+^CD62L^low^	Human	“Aged” cell	Regression into the bone marrow	170
	Mouse			
		Breast cancer	Vital NETosis Promote metastasis	171
CD11c^bright^CD62L^dim^CD11b^bright^ CD16^bright^	Human	Experimental endotoxemia	Immunosuppression	172
CD177^+^	Human	Pregnancy	Facilitates endothelial transmigration	173
		Sepsis		174
		Periodontitis Arthritis		175
		Vasculitis Systemic lupus erythematosus	Autoimmunity	176
CXCR4^high^CD11B^high^CD62L^low^ CCR2^low^	Mouse	Skin infection	Migration to lymph nodes	81
CD54^high^CD18^high^CD62L^low^ CXCR1^low^CXCR2^low^	Human	Systemic inflammation	Reverse transendothelial migration	72
OLFM4^+^	Mouse	Healthy	Colocalizes with NETs	177
		Hemorrhagic Shock	Portend poor prognosis	178
	Human			
		Sepsis	Portend poor prognosis	179
	Human	Vasculitis	Autoimmunity	180
VEGFA^+^ARG1^+^CCL2^+^	Human	Pregnancy	Promote angiogenesis	181
CD49d^+^VEGFR1^high^CXCR4^high^	Human	Hypoxia		182
	Mouse			
CD11b^+^Gr-1^+^CXCR4^high^	Mouse	Transplant/Hypoxia		183
MMP-9^+^HIF-1α^+^	Human	Nasal inverted papilloma		184
CD66b^+^CD10^+^	Human	G-CSF–treated donors	Inhibit T cells	185
CD66b^+^	Human	Sepsis	Immunosuppression	27
CD16^high^CD62L ^low^	Human	Experimental endotoxemia	PD-L1 mediated immunosuppression	186
PD-L1^+^	Mouse	*Candida albicans* infection	Neutrophil accumulation in the bone marrow	187
	Human			
		Sepsis	Increased neutrophil lifespan	188
		Rheumatoid arthritis	Associated with disease severity	189
		COVID-19		
PMN-MDSCs CD11b^+^CD14^−^CD15^+^/CD66b^+^ CD15^+^/CD66b^+^CD14^−^LOX1^+^ CD15^+^/CD66b^+^CD14^−^CD84^+^	Human	Pregnancy Autoimmune diseases Cancer Severe COVID-19	Inhibit T, B, and NK cells	190
PMN-MDSCs CD11b^+^Ly6G^+^Ly6C^low^ CD11b^+^Ly6G^+^CD84^+^	Mouse			
Low-Density Neutrophils	Human	Pregnancy Sepsis Diabetes Cancer	Immunosuppression	191 – 195
	Human	Systemic lupus erythematosus	Increased NET formation	196, 197
TAN-N1 SiglecF^+^	Mouse	Lung cancer	Tumor killing	198 – 201
TAN-N2	Mouse	Lung cancer	Tumor promoting	198, 199
CD15^null/low^CD16^null/low^CD11b^high^	Human	Neutropenia	Reduced antibody production	41
HLA-DR^+^CD86^+^	Human	Lung cancer	Antigen presentation Trigger antitumor T-cell response	202
CCL4^+^	Human	Liver cancer	Macrophage recruitment	203
CD10^+^ALPL^+^	Human	Hepatocellular carcinoma	“Irreversible” exhaustion of T cells	204
CD11b^high^CD54^high^CD62L^low^	Mouse	Sarcoma	Immune resistance	205

NET, neutrophil extracellular traps; NK cells, natural killer cells; PD-L1, programmed death ligand 1; PMN-MDSCs, polymorphonuclear neutrophil-myeloid-derived suppressor cells; TAN-N1, tumor-associated neutrophil subset N1.

Numerous neutrophil subsets have been described in pathogen-associated inflammation (Table 1). For example, human experimental endotoxemia triggers a subset of mature hypersegmented neutrophils, characterized as CD11c^bright^CD62L^dim^CD11b^bright^ CD16^bright^, with immunosuppressive activity (172) through expression of arginase-1 (209) and programmed death ligand-1 (PD-L1) (186, 210). SARS-CoV-2 and rheumatoid arthritis were also reported to drive PD-L1^+^ neutrophils (189). PD-L1^+^ neutrophils have been implied to contribute to increased susceptibility to infection following tissue injury (211) and liver metastasis in pancreatic cancer (212).

Low(er)-density neutrophils refer to a heterogenous subset that co-segregates with mononuclear cells after density-gradient isolation (213), compromising both immature (banded nucleus) and hypersegmented neutrophils (214). It is uncertain whether this subset represents a distinct lineage of cells or the neutrophil activation state in disease (213). Indeed, increased numbers of low-density neutrophils have been detected during normal pregnancy (191), sepsis (192), diabetes (193), autoimmune diseases (196), and cancer (194, 195). Low-density neutrophils may exert immunosuppression function, hence also known as granulocytic myeloid-derived suppressor cells (196), and have a higher capacity of bacterial containment than high-density mature neutrophils (213). Contrasting their immunosuppressive function, low-density neutrophils have also been implicated in the pathogenesis of systemic lupus erythematosus through exacerbated production of type I interferons and release of NETs (196, 197). An intriguing possibility is that low-density neutrophils are cells that underwent reverse transendothelial migration and acquired the activation phenotype within the tissue (72).

Distinct neutrophil populations, a proinflammatory subset, displaying potent tumor-killing capacity (termed N1), and an anti-inflammatory tumor-promoting (N2) phenotype have been described (198, 199). The anti-inflammatory cytokine-transforming growth factor-β (TGF-β) and IFN-β have been implied in polarizing tumor-associated neutrophils toward an antitumorigenic and antiangiogenic phenotype (215, 216). N1 neutrophils possess increased arginase activity (198) and express the sialic acid-binding protein SiglecF (200, 201). SiglecF^+^ neutrophils exhibited an extended lifespan (6–8 days) within the lung tumor microenvironment (201, 217). High-resolution single-cell transcriptomics of human and mouse nonsmall cell lung cancers revealed six distinct resident myeloid populations that are conserved across individuals and species (218, 219). The origin and functions of these subsets and the link to the N1/N2 paradigm are not known. In a cancer environment, protumorigenic neutrophils might be released from the bone marrow and home in the lung (53), a process that likely requires expression of the proto-oncogene *Met* (220).

## NEUTROPHILS IN TISSUE REPAIR

**Figure 2.**
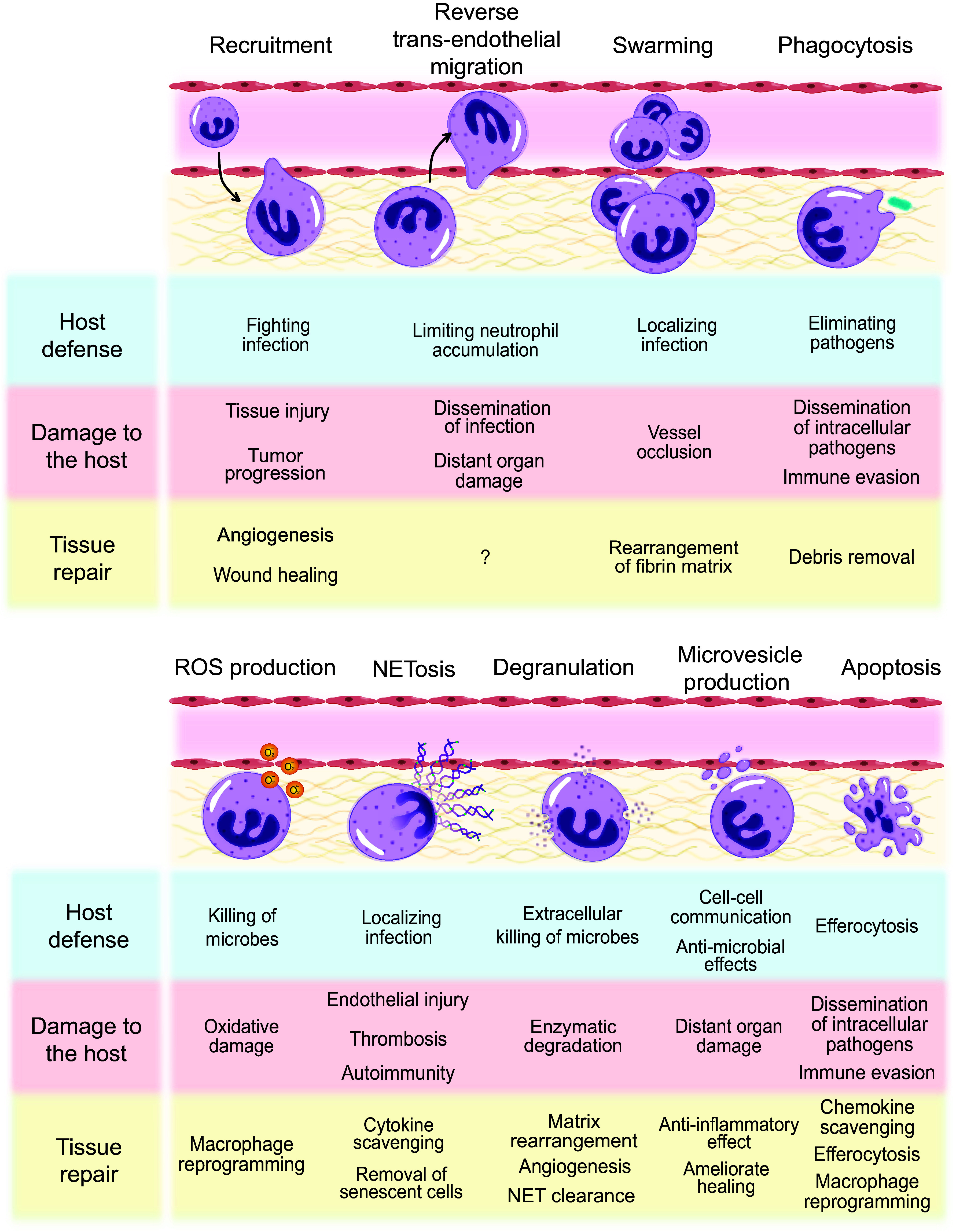
Neutrophil functions in host defense, tissue damage, and repair. Neutrophil trafficking into tissues is essential for host defense, but can also lead to tissue damage and may promote tumor progression. Neutrophils are also required for wound healing and angiogenesis. Neutrophil may egress from the tissue through reverse transendothelial migration that may limit neutrophil accumulation or contribute to dissemination of infection and distant organ damage. Neutrophils swarm toward pathogens to localize infection and to form a tight wound seal, whereas intravascular swarming may result in occlusion of blood vessels. Swarming can also contribute to rearrangement of fibrin matrix. Phagocytosis leads to elimination of pathogens or damaged cells, but may contribute to dissemination of intracellular pathogens and immune evasion. Phagocytosis clears debris, an essential step of the initial scar formation to prepare the infected/injured tissue for repair. Phagocytosis usually triggers apoptosis in neutrophils followed by efferocytosis and reprogramming of macrophages toward a proresolution phenotype that mediates repair. Production of reactive oxygen species (ROS) contributes to killing of microbes, collateral tissue damage, and macrophage reprogramming. Release of neutrophil extracellular traps (NETs) localizes infection, may demarcate the injured area, while excessive NETosis may evoke endothelial injury, thrombosis, and autoimmunity. Release of granule contents contribute to extracellular killing of microbes and granule enzymes are essential components of NETs. Proteolytic enzymes degrade tissues and perpetuate injury. Granule constituents play important roles in rearranging the extracellular matrix, inducing angiogenesis and facilitating clearance of NETs by macrophages. NETs can scavenge cytokines and mediate removal of senescent cells. Neutrophil-derived microvesicles ensure cell-cell communications and may exert protective and deleterious effects in content and context-dependent fashion. Thus, microvesicles may exert antimicrobial actions, contribute to distal organ damage or dampen inflammation, and ameliorate healing. Neutrophils integrate prosurvival and apoptosis-promoting cues from the inflammatory microenvironment, which governs their life span. Apoptotic neutrophils are removed by macrophages via efferocytosis that is associated with macrophage polarization toward the proresolution phenotype and secretion of mediators that promote repair. Apoptotic neutrophils carrying intracellular pathogens that they cannot destroy may function as “Trojan horses” to infect macrophages and contribute to immune evasion. Apoptotic neutrophils express CCR5 (C-C chemokine receptor type 5), which by binding chemokines may prevent excessive neutrophil recruitment.

Inadvertent and perpetual neutrophil activation leads to nonresolving inflammation and persisting tissue injury, a critical component of numerous pathologies. This would explain the beneficial effects of prophylactic neutrophil depletion in reducing the extent of tissue damage as observed in numerous studies, however, the majority of these studies did not assess tissue repair and return to homeostasis, the ideal outcome of the inflammatory reaction (4). Accumulating data challenge the simplistic view that associates the prolonged presence of neutrophils with tissue injury and indicate a seminal role for neutrophils in the resolution of inflammation and repair (Fig. 2). Indeed, by processing debris in the damaged areas, neutrophils prepare these areas for tissue regeneration (221). For instance, neutrophil-borne matrix metalloproteinases (MMPs) modulate matrix-cell and cell-cell interactions through cleavage of structural proteins such as collagen of the extracellular matrix (222). This would lead to loosening of cell-matrix attachments, allowing cell migration that is required for re-epithelization (223). Concordantly, addition of MMP2 or MMP9 improved wound closure rates in conventional in vitro assays (224, 225). Moreover, treatment of neutropenic mice with MMP9 was reported to enhance in vivo epithelial repair in the lung (226). Neutrophil elastase facilitates the deposition of collagen (75) and induces fibroblast proliferation and myofibroblast differentiation (227), indicating a profibrotic role. Scar formation occurs at an early stage following tissue injury and serves as a temporary support for injured tissue as well as a template for subsequent cell repopulation (221). Neutrophil-mediated remodeling of fibrotic areas was observed in muscles after injurious stretch (228) and in skin wounds in mice (229). Conversely, neutropenia was associated with delayed re-epithelialization and decreased compensatory cell proliferation in mouse models of acute lung injury (230, 231). There is evidence that sprouting blood vessels attract angiogenic VEGFR1^+^ neutrophils, which through the release of MMP9 promote neovascularization (232) as observed in transplants (182, 232), the postinfarct heart (233), and tumor microenvironment (234, 235). Inhibiting recruitment of VEGFR1^+^ neutrophils resulted in reduced vascular density (232). These observations underscore the contribution of neutrophils to successful tissue regeneration by preparing the “soil for seeding” of stem cells and other cells with reparative potential (221).

Another mechanism by which neutrophils contribute to tissue repair is activation of macrophage-centered feed-forward proresolution programs. Phagocytosis of apoptotic neutrophils by macrophages leads to the release of TGF-β and interleukin-10 (IL-10), which promote tissue repair (143, 144) and interferon-β (IFN-β) (112). IFN-β enhances neutrophil phagocytosis (112, 236), apoptosis, and efferocytosis (112, 237, 238), and reprograms macrophages to the CD11b^low^ resolution phenotype (112). The actions of IFN-β are, at least in part, mediated by triggering the synthesis of preresolving lipid mediators, such as 15-epi-LXA_4_ and resolvin D1, which signals through ALX/FPR2 (238). Resolution-phase macrophages were found to accumulate and fragment lactoferrin, an antibacterial protein stored in the specific granules in neutrophils (239). Upon release, lactoferrin fragments stimulate macrophage reprogramming and the formation of resolution-promoting aggregated NETs (239, 240). In an experimental model of gout, aggregated NETs limited neutrophil recruitment and activation by degrading cytokines and chemokines through serine proteases (240).

While these mechanisms appear to be nonspecific and likely occur in all organs, the neutrophil response is often time-dependent (75) and will likely be context-dependent to adapt to the differences in organ architecture and specific cell types to be repaired. Below, we discuss neutrophil-mediated effector functions in tissue repair in different organs.

### Repair of Lung Injury

The lung houses a neutrophil population that possesses unique phenotypes and functions. At steady- state, neutrophils accumulate intravascularly within the pulmonary microvasculature, forming the marginated pool (241–244). This pool is maintained by CXCR4*/*CXCL12 signaling (244). Marginated neutrophils crawl along the capillary walls (242, 243) and rapidly phagocytose bacteria retained by the lung endothelium (243). Furthermore, pulmonary neutrophils have been shown to express the angiogenic factors *Apelin* and *Vegfa* and to promote the proliferation of pulmonary endothelial cells, a characteristic feature of lung development (165).

Excessive recruitment and activation of neutrophils have long been recognized as a pathogenetic factor in the onset and progression of acute lung injury as well as a predictor of disease severity and outcome (226). Consistently, prophylactic depletion of neutrophils or pharmacological blockade of neutrophil recruitment or function protected against lung damage in various experimental models, including transfusion-induced acute lung injury (245) and ventilator-induced lung injury (246). By contrast, neutropenic patients have worse outcomes of acute lung injury (10), and interventional trials aimed at reducing neutrophil recruitment or activation showed no clinical benefit at 28 day and even was associated with an increase in 180-day all-cause mortality as reported by the STRIVE trial (inhibition of neutrophil elastase with Sivelastat) (247).

Persisting presence of neutrophils was observed during lung repair in murine models with surviving animals exhibiting more pronounced neutrophilic infiltrates (224). Depletion of neutrophils 24 h after inducing pulmonary injury delayed re-epithelialization and recovery in models of acid instillation (230) and ventilator-associated lung injury (226). Results from these models coupled with those from cellular and clinical studies linked epithelial regeneration to sustained activity of neutrophil-derived MMP9, and, to a lesser degree, MMP2 and Fgf1 (226, 230). Concordantly, treatment of neutropenic mice with MMP9 markedly reduced pulmonary damage, indicating enhanced repair (231). Moreover, exogenous MMP9 restored the repair potential of bronchoalveolar lavage fluid from neutropenic patients in an ex vivo model of alveolar injury (231).

Within the injured zone, neutrophils clear all debris and prepare the damaged areas for repair (221). Neutrophils are the predominant source of MMPs, in particular MMP9, within the injured lung, which exert multifaceted actions, including modulation of cell-matrix and cell-cell interactions through cleavage of structural proteins, such as collagen (222, 248). MMP9 facilitates neutrophil egress into the pulmonary parenchyma in response to Toll-like receptor (TLR)-induced MyD-88-mediated release of chemotactic factors, forming a feed-forward loop for neutrophil recruitment (249). At this stage, neutrophil-borne MMP9 and neutrophil elastase facilitate deposition of collagen and contribute to neovascularization of the damaged tissue (75). MMP9 is secreted as a latent proenzyme that requires activation to expose its catalytic site. MMP9 activators include neutrophil elastase and proteinase 3 (85). Activated MMP9 evokes the release of proangiogenic factors such as VEGF and fibroblast growth factors (250). Neutrophil elastase was shown to induce both fibroblast proliferation and myofibroblast differentiation, implying a profibrotic role in the initial scar formation (227). Moreover, these proteases can also facilitate the resolution of inflammation by degrading proinflammatory cytokines (251), alarmins (252), and DAMPs such as high-mobility group box 1 (HMGB1) and heat shock protein 90 (HSP90) (253), thereby disrupting the recruitment of additional inflammatory cells.

Neutrophil transmigration can itself activate epithelial repair through activating the Wnt/β-catenin pathway, likely via neutrophil elastase-mediated cleavage of E-cadherin, in TM4SF1^+^ alveolar type 2 epithelial cell population (254). The Wnt-responsive alveolar epithelial progenitor lineage differs from other proposed lung progenitor cells, exhibits distinct transcriptome and epigenome, and expands rapidly after acute lung injury (255). Wnt signaling leads to the release of Cyr61 (cysteine-rich angiogenic inducer 61), which promotes epithelial repair (256). Another mechanism by which neutrophils may attenuate lung damage is transfer of microRNA-223 from neutrophils to pulmonary epithelial cells, leading to suppression of poly(ADP-ribose) polymerase 1 (PARP-1) (257).

### Cardiovascular Repair

Neutrophils rapidly accumulate in the ischemic myocardium and are sustained for over 7 days after infarction (258). The prevailing view has been that recruited neutrophils through the release of NETs, granular enzymes, and the alarmins S100 calcium-binding protein A8 and A9 (S100A8/A9) aggravate the initial myocardial damage and promote pathological remodeling in the ventricular wall (259, 260). S100A8/A9 also stimulates granulopoiesis in the bone marrow, forming a feed-forward loop for neutrophil trafficking into the ischemic myocardium (261, 262). However, recent data suggest a protective role for neutrophils. Thus, chronic antibody-mediated neutrophil depletion was reported to further worsen cardiac function, leading to heart failure and increased cardiac fibrosis in a mouse model of myocardial infarction (263). Recruited neutrophils spread on necrotic tissues that initiate phagocytosis of injured cardiomyocytes and cell debris and subsequently promote neutrophil apoptosis (264). Dying neutrophils secrete α-defensins, which enhance efferocytosis, dampen the release of proinflammatory mediators (265), and sequester chemokines through expression of CCR5 (C-C chemokine receptor type 5) (266), thereby limiting the inflammatory reaction. Phagocytosis of apoptotic neutrophils polarized macrophages toward a reparative phenotype (Ly6C^low^MerTK^high^) 3 days after myocardial infarction (267), which was mediated by neutrophil gelatinase-associated lipocalin (NGAL) (263) and S100A9 (268). Consistently, treatment with recombinant NGAL induced reparative macrophages in neutrophil-depleted mice, whereas long-term S100A9 blockade recapitulated the deleterious actions of neutrophil depletion on recovery from myocardial infarction (263). These findings also underscore the importance of timing to inhibit S100A9 to prevent its deleterious effects without hindering its beneficial actions. Other studies reported an increased abundance of N2 repair-like neutrophils, characterized by phenotypic and transcriptomic changes, within the infarct zone (269). These neutrophils, together with macrophages secreted oncostatin M, which stimulated cardiac fibroblasts and cardiomyocytes to promote angiogenesis (270, 271). NET formation was suggested to modulate fibrotic remodeling after myocardial infarction in patients, possibly through stimulating fibrocytes (272). Neutrophil-released cathelicidin (human LL-37, mouse CRAMP) promoted re-endothelialization after acute injury in mice and patients (273). Another mechanism, TNFα-mediated activation of the CCL20 (C-C motif chemokine ligand 20)-CCR6 (C-C chemokine receptor type 6) axis was shown to recruit VEGFA-expressing neutrophils to sites of injury to initiate revascularization following femoral artery ligation in mice (274). Future research should reveal the origin of repair-like neutrophils and the stimuli for neutrophil reprogramming.

### Liver Repair

Neutrophil infiltration and activation are common events underlying most forms of acute and chronic liver diseases (275). Compelling evidence indicates that hepatic neutrophils also serve as resolving effector cells and contribute to the remarkable regenerative capacity of the liver. Indeed, neutrophil depletion was reported to augment cellular debris and delay revascularization and repair in sterile thermal hepatic injury (75) and acetaminophen-induced acute liver injury in mice (276). In this latter study, the detrimental effects were observed when neutrophil depletion was performed during the repair phase, contrasting with reduced hepatic necrosis observed with prophylactic neutrophil depletion during the early phase of acetaminophen poisoning (276). Neutrophil depletion during the resolution phase resulted in diminished macrophage phenotypic switch to a regenerative profile and defective repair processes in diet-induced murine steatohepatitis (277). Recent studies identified neutrophil-derived ROS (278, 279) and microRNA-223 (miR-223), a negative regulator of NOD-like receptor 3 (NLRP3) expression (280), as important mediators that trigger the generation of reparative Ly6C^low^ CXCR3^high^ macrophages that orchestrate the resolution of inflammation and liver repair. Subsequent studies documented the beneficial effects of treatment with the synthetic miR-233 analog miR-223 3p in models of acute and chronic liver injuries (281), underscoring the translational potential of this approach. Neutrophil-borne cathelicidin (LL-37) also promoted repair of acetaminophen-induced liver injury in mice, possibly through enhancing neutrophil phagocytosis (282). Clearance of hepatectomy-induced apoptotic extracellular vesicles stimulated neutrophils to release various growth factors, such as fibroblast growth factor-2 (FGF-2) and hepatocyte growth factor (HGF), which have been implied in liver regeneration (283). Neutrophil-secreted MMP8 and MMP9-mediated fibrinolysis suppressed fibrosis in CCl_4_-induced chronic liver injury in mice (284), and MMP8 facilitated collagen resorption during repair of cholestatic rat livers (223).

### Skin Wound Healing

Neutrophil accumulation in the injured skin is generally associated with efficient cutaneous wound repair presumably through containing invading pathogens (285). Patients with neutropenia or deficiencies in phagocytosis often suffer from delayed wound healing (136). Mice deficient of CXCR2 (286) or formyl peptide receptor 1 and 2 (Fpr1/2) (287) exhibited reduced neutrophil infiltration and delayed wound closure. It is noteworthy that the effects of Fpr1/2 deletion are not restricted to modulating neutrophil trafficking, for these promiscuous receptors bind multiple ligands, many of which are also involved in orchestrating neutrophil-centered proresolution circuits (49, 60, 288, 289). Neutrophil depletion was associated with impaired wound healing in aged, but not young mice (229). Treatment with G-CSF restored this defect (229), suggesting differences in neutrophil generation during aging. Upon recruitment, neutrophils through the release of MMP8 evoked degradation of the extracellular matrix and generated the chemotactic peptide proline-glycine-proline, begetting recruitment of additional neutrophils (290). Recruited neutrophils orchestrate the reparative cascade, including CXCL-10-mediated recruitment of plasmacytoid dendritic cells that have a high capacity to clear commensal microbes (291). NET components, such as chromatin, histone, and myeloperoxidase were shown to trigger wound-healing programs in macrophages, fibroblasts, and keratinocytes (292, 293). In contrast, neutrophils from diabetic mice readily undergo NETosis, which contributes to the well-documented delay in wound healing in diabetes (193, 294). The phenotype and fate of cutaneous neutrophils remain largely unexplored.

### Bone Fracture and Skeletal Muscle Healing

The role of neutrophils in bone fracture healing is well recognized, though excessive neutrophil influx has been suggested to explain poor healing in polytrauma (295–297). Neutrophils infiltrate the initial fracture hematoma and contribute to synthesis of fibronectin. This “emergency extracellular matrix” (298) serves as a scaffold for immune cells and stromal cells (295). N2-polarized neutrophils were suggested to guide the recruitment of bone mesenchymal stem cells and initiate bone regeneration (299). There is evidence that neutrophil-derived microvesicles can also modulate regenerative capacity. In a heterochronic parabiosis model, Ly6G^+^ microvesicles generated in young mice were found to ameliorate fracture healing in aged mice (300). Neutrophil-derived microvesicles expressing AnxA1 can penetrate cartilage, and through interaction with its receptor FPR2 stimulate TGF-β production (339). Microvesicle-expressed AnxA1 and phosphatidylserine prevented activation of inflammatory M1 macrophages in rheumatoid arthritis (156), suggesting therapeutic potential for neutrophil-derived microvesicles.

In skeletal muscle stretch injury models, nonselective depletion of neutrophils and monocytes with antisera or blockade of Mac-1 resulted in the accumulation of more tissue debris (302) and impaired initial regenerative response (228). It is uncertain whether these protective actions can solely be attributed to neutrophils. Recent data suggest that natural killer cells antagonize neutrophil accumulation via inducing apoptosis and C-C chemokine receptor type 1 (CCR1)-mediated chemotaxis parallel with TGF-β-mediated impairment in muscle stem cell regenerative function in a mouse model of degenerative volumetric muscle loss injury (303). These findings underscore the importance of immune cell-stem cell cross talk in driving regeneration in skeletal muscle, which should be a topic for future investigations.

### Repair of Nervous Tissues

The adult central and peripheral nervous system possesses limited capacity for self-repair following injury. Neutrophils are traditionally considered detrimental and unfavorable to tissue regeneration in the nervous system (304). Recent data challenge this view and propose a beneficial role for neutrophils and inflammation in neuroprotection, axonal regeneration, and functional recovery (301, 305). Neutrophils were shown to replace CCR2^+^ macrophages as the primary phagocytic cells and initiate neuronal repair by clearing myelin debris via phagocytosis following peripheral nerve injury (306). In a model of optic nerve injury, neutrophils were identified as a major source of oncomodulin, a neurotrophic factor that promotes optic nerve regeneration (307). Neutrophil depletion or an oncomodulin peptide antagonist prevented optic nerve repair, whereas macrophages were insufficient to stimulate regeneration in the absence of neutrophils (307), further underscoring the importance of neutrophils driving neuronal repair. Neutrophil depletion in mice also delayed functional recovery from spinal cord injury and delayed astrocyte reactivity, indicating neutrophil modulation of glial function (308). However, it is uncertain whether the neuroprotective action can solely be attributed to neutrophils, for the anti-Ly6G/Gr-1 antibody employed in this study depletes both neutrophils and monocytes. Neutrophils and astrocytes in the spinal cord can release secretory leukocyte protease inhibitor (SLPI) that was shown to improve locomotor control and reduce secondary tissue damage following spinal cord injury in mice (309). Furthermore, human and mouse bone marrow neutrophils polarized with a combination of IL-4 and G-CSF produced an array of growth factors that induced neurite outgrowth (310). Adoptive transfer of these polarized neutrophils triggered axon regeneration within the optic nerve and spinal cord in murine models (310).

### Regeneration in the Eye

Beyond their paradigm proinflammatory role, unconventional functions for neutrophils have been reported in the eye. Resident tissue neutrophils in the cornea have been reported to mediate healing of minor epithelial injuries and drive nerve regeneration (311–314). Corneal limbus and lacrimal gland neutrophils produce the proresolving lipid mediator LXA_4_ that amplifies regulatory T cells (T_reg_) and inhibits effector T-helper cell 1 (Th1) and Th17 cells (315). Desiccating stress resulted in the loss of neutrophils and LXA_4_ parallel with marked increases in Th1 and Th17 cells and decreases in T_reg_ in female mice only, leading to dry eye pathogenesis (315). These findings identified female-specific regulation of corneal neutrophils as a key factor in preventing aberrant T-cell activation and initiation of autoimmune dry eye disease. Subsequent studies showed that docosahexaenoic acid-enriched diet upregulated LXA_4_-producing corneal neutrophils and protected against development of dry eye disease in mice (316). Intriguingly, neutrophil and Th17 cell accumulation was detected in the conjunctival sac following 7 h sleep (317), though the biological importance of these observations is not known. Studies on a mouse model of ischemic retinopathy and patients with proliferative diabetic retinopathy revealed an endogenous repair mechanism centered on the attraction of neutrophils to the senescent vasculature followed by extrusion of NETs onto diseased vessels (318). These target the senescent vasculature for clearance and prepare the damaged vessels for reparative remodeling (318).

### Neutrophils in Solid Tumors

In 1986, Harold Dvorak coined the phrase that cancer is a “wound that does not heal” (319), implying similarities in the cellular and biochemical processes underlying wound healing and development of tumor stroma. Neutrophils infiltrate many solid tumors. For instance, neutrophils are the most prevalent immune cells in nonsmall cell lung cancer, where high numbers of infiltrating neutrophils and a high neutrophil-to-lymphocyte ratio portend poor prognosis for patient survival (320). Tumor-associated neutrophils exhibit diverse context-dependent behaviors (321), which have been attributed to their dual functions as proposed by the N1 (antitumorigenic) and N2 (protumorigenic) paradigms (198). In the tumor microenvironment, TGF-β was found to polarize neutrophils toward the N1 phenotype (198). N1 neutrophils can kill tumor cells directly or through participating in the cellular network that mediates antitumor resistance (322). Intriguingly, ROS produced by tumor-infiltrating neutrophils has been reported to deplete IL-17-producing γδ17 T cells in mice, resulting in an immunosuppressive yet antitumoral microenvironment (323). The N2 neutrophil subset creates a favorable environment for tumor progression through the production of ROS and angiogenic factors, enabling the growth of the tumor vasculature during the early stages of carcinogenesis in mouse models (215, 234). Treatment of protumor CD11b^ + ^Gr1^+^ neutrophils with IFN-β reduced the expression of proangiogenic factors to control levels (215), suggesting a role for constitutively produced endogenous IFN-β in innate tumor surveillance. Furthermore, N2 neutrophils suppressed the proliferation and activity of cytotoxic T lymphocytes through the secretion of arginase 1 and ROS (324) and upregulation of PD-L1 (186, 210). Studies in invasive breast cancer showed that tumor cells can reprogram early myeloid differentiation in the bone marrow to generate immunosuppressive neutrophils (325). N2 neutrophils have been reported to support the spread of metastases to distant organs (212, 326, 327). One should note the complex interplay between tumor-associated neutrophils and tumor progression as exemplified by a neutrophil subset with features of antigen-presenting cells, which triggered an antitumor T cell response in early-stage (small size) human lung cancer, whereas this subset was undetectable in larger tumors (202).

## NEUTROPHIL-TARGETING THERAPEUTIC STRATEGIES: WHAT THE FUTURE HOLDS

Considering the multifaceted roles of neutrophils in homeostasis, inflammation, and repair, it is paramount to seek novel therapeutic approaches controlling neutrophil-mediated collateral tissue damage and/or enhancing their reparative and/or regenerative potential. A simple “one size fit all” antineutrophil approach is naïve and outdated (328). Universal targeting approaches aimed at reducing neutrophil numbers carriying the risk of increased susceptibility to bacterial and fungal infections. Over the past years, more-selective strategies have been developed, including β_2_ integrin antagonists, inhibitors of degranulation and NETosis, and NET-degrading agents, which showed promising results in preclinical models to prevent the detrimental effects of neutrophils (329–332). Some compounds have already been tested in patients [for example, CXCR2 antagonists to block neutrophil chemotaxis in cystic fibrosis (333) and a neutrophil elastase inhibitor in acute lung injury (247)] with limited success. We argue that subtype-selective targeting strategies, i.e., depleting or inhibiting harmful neutrophil subsets while preserving beneficial subtypes or harnessing their reparative potential would be more desirable. However, such an approach is hindered by our limited knowledge to identify the phenotype of these subsets. Furthermore, it is still unclear whether the multifaceted actions are mediated by different polarization states of mature neutrophils or distinct neutrophil subsets. A major challenge is to understand how neutrophils develop reparative functions and whether all neutrophils harbor these functions. It is still unknown whether distinct neutrophil subsets are recruited during the initial and resolution phases of the inflammatory reaction, as suggested by studies in myocardial infarction (260) or whether recruited neutrophils are reprogrammed to execute repair functions within the inflammatory microenvironment, as suggested by the N1/N2 paradigm in solid cancers (198, 201, 217). Future studies should address tissue-specific cues that shape the expression of diverse repair programs.

An interesting therapeutic approach, representing a conceptual change for the treatment of inflammatory pathologies, is to harness FPR2 agonists to shift the balance toward resolution. The intriguing biology of the ALX/FPR2 receptor has initiated several medicinal chemistry programs to develop small-molecule agonists to activate resolution programs (288, 334, 335). Relevant examples here are the beneficial actions of synthetic lipoxin mimetics (336) and the peptide agonist WKYMVM in cardiac repair in mice (337). Translation of these promising findings to the clinical setting is, however, challenging because of many differences between human and murine neutrophil biology (338).

## CONCLUDING REMARKS

The development of novel technologies and animal models have contributed to recognition of the phenotypic heterogeneity and functional plasticity of neutrophils. The traditional view of neutrophil dichotomy, antimicrobial defense functions versus fueling inflammation, does not account for all aspects of neutrophil biology. Indeed, neutrophils are increasingly recognized as effectors of resolution and tissue repair. Whether the opposing functions are mediated by distinct bona fide neutrophil subsets or different polarization states of mature neutrophils remains to be explored. Although challenging, linking neutrophil phenotypes to distinct functions will be essential to improve current therapies. This ongoing research highlights the importance of the healing power of neutrophils and will likely spur further advances in neutrophil-targeted therapies to dampen inflammation and favor reparative processes without compromising antimicrobial host defense.

## GRANTS

This work was supported by grants from the Canadian Institutes of Health Research (MOP-102619 and PJT-169075) (to J.G.F.).

## DISCLOSURES

No conflicts of interest, financial or otherwise, are declared by the authors.

## AUTHOR CONTRIBUTIONS

S.A.R-T. and J.G.F. conceived and designed research; prepared figures; drafted manuscript; edited and revised manuscript; and approved final version of manuscript.
